# Learning course adjustments during arm movements with reversed sensitivity derivatives

**DOI:** 10.1186/1471-2202-11-150

**Published:** 2010-11-26

**Authors:** Mohamed N Abdelghani, Douglas B Tweed

**Affiliations:** 1 Department of Physiology, University of Toronto, Toronto, Canada; 2 Department of Medicine, University of Toronto, Toronto, Canada; 3 Centre for Vision Research, York University, Toronto, Canada

## Abstract

**Background:**

To learn, a motor system needs to know its sensitivity derivatives, which quantify how its neural commands affect motor error. But are these derivatives themselves learned, or are they known solely innately? Here we test a recent theory that the brain's estimates of sensitivity derivatives are revisable based on sensory feedback. In its simplest form, the theory says that each control system has a single, adjustable estimate of its sensitivity derivatives which affects all aspects of its task, e.g. if you learn to reach to mirror-reversed targets then your revised estimate should reverse not only your initial aiming but also your online course adjustments when the target jumps in mid-movement.

**Methods:**

Human subjects bent a joystick to move a cursor to a target on a computer screen, but the cursor's motion was reversed relative to the joystick's. The target jumped once during each movement. Subjects had up to 4000 trials to practice aiming and responding to target jumps.

**Results:**

All subjects learned to reverse both initial aiming and course adjustments.

**Conclusions:**

Our study confirms that sensitivity derivatives can be relearned. It is consistent with the idea of a single, all-purpose estimate of those derivatives; and it suggests that the estimate is a function of context, as one would expect given that the true sensitivity derivatives may vary with the state of the controlled system, the target, and the motor commands.

## Background

To learn effectively, a motor system needs to know how its error ***e ***(for instance, the vector from target to hand in a reach) depends on the vector of neural commands ***u ***sent to the muscles (e.g. the signals to biceps, triceps, brachioradialis etc.). Mathematically, what the system needs is the matrix ∂***e***/∂***u***, called the control jacobian [1] or the matrix of *sensitivity derivatives *[2]. But is ∂***e***/∂***u ***itself learned, or is it known innately? Here we test a recent theory which holds that the brain's estimates of sensitivity derivatives are not solely innate but are deduced from sensory feedback [3].

Abdelghani et al. [3] pointed out the importance, for this question, of the *signs *of the elements of ∂***e***/∂***u***: if your brain knew ∂***e***/∂***u ***innately, then over time its innate estimates would of course become inaccurate (owing to your growth, aging, injuries, and healing), but so long as the signs of the estimates were correct, you could usually maintain good performance. But if the signs of ∂***e***/∂***u ***reversed (e.g. if you put on reversing goggles and tried to reach for things) then your innate estimates would make you "learn" the wrong way, strengthening those components of ***u ***that should be weakened and vice versa. Given this kind of reversal, recovery is possible only for systems that can revise their estimates of ∂***e***/∂***u***. In [3] we argued that neural controllers can learn this kind of task, and we proposed a mechanism, called *implicit supervision*, by which the brain might deduce ∂***e***/∂***u***.

There is empirical support for implicit supervision. It is the only theory that explains how neural controllers can deal with sign changes in ∂***e***/∂***u***, as happen with reversed vision or nerve transposition [4-14]. And it explains why it is harder to adapt to reversals than to other changes [3,15,16].

The next question is whether adjustable estimates of sensitivity derivatives govern all aspects of a task. For instance, when you learn to move to mirror-reversed targets, does your adapted estimate of ∂***e***/∂***u ***reverse both your initial aiming and your online course adjustments: when the target jumps in mid-movement, is your path adjustment appropriately reversed?

Data relevant to this issue have come from a novel experiment by Gritsenko and Kalaska [17]. They trained people to reach to stationary (i.e. non-jumping) right-left-reversed targets. After the training was complete, they tested the subjects' responses when the mirror-reversed target jumped suddenly in mid-reach, and they found that in many cases the subjects' earliest course adjustments were *not *appropriately reversed, as they should have been if ∂***e***/∂***u ***had been learned. What are the implications of this fact for the theory of implicit supervision? Does it mean that the reach controller in the brain has multiple estimates of ∂***e***/∂***u ***-- one perhaps concerned with launching a reach toward its target, and a different one concerned with course adjustments? Is this latter estimate incapable of adapting, or might it adapt given a different training regimen -- the point of Gritsenko and Kalaska's study was to train on stationary targets and then test generalization to jumps, but what if subjects were trained on jumping targets? And finally, might Gritsenko and Kalaska's findings be compatible after all with a single, all-purpose estimate of ∂***e***/∂***u ***rather than separate ones for launch and adjustment?

Here we put subjects through many trials with jumping targets, and show that they can learn to reverse their rapid, online course adjustments; i.e. we show that these adjustments are governed by an adjustable estimate of ∂***e***/∂***u***. And we argue that all the available data are compatible with a single, adaptable, all-purpose estimate.

## Methods

This study complies with the Helsinki Declaration and was approved by the Ethics Review Office of the University of Toronto, reference number 16210. All subjects gave their informed consent.

### Experimental task

Subjects bent a joystick to move a cursor toward a jumping target on a computer screen. They sat facing the screen at a distance of 80 cm, and used their dominant arm to manipulate an Impulse Stick -- a USB force feedback joystick made by Immersion Inc. (San Jose, CA, USA) -- through its full range of ±40°, or about ±6 cm. The joystick was placed to the subject's right or left side, its *x*-axis parallel with the screen.

**Figure 1:**
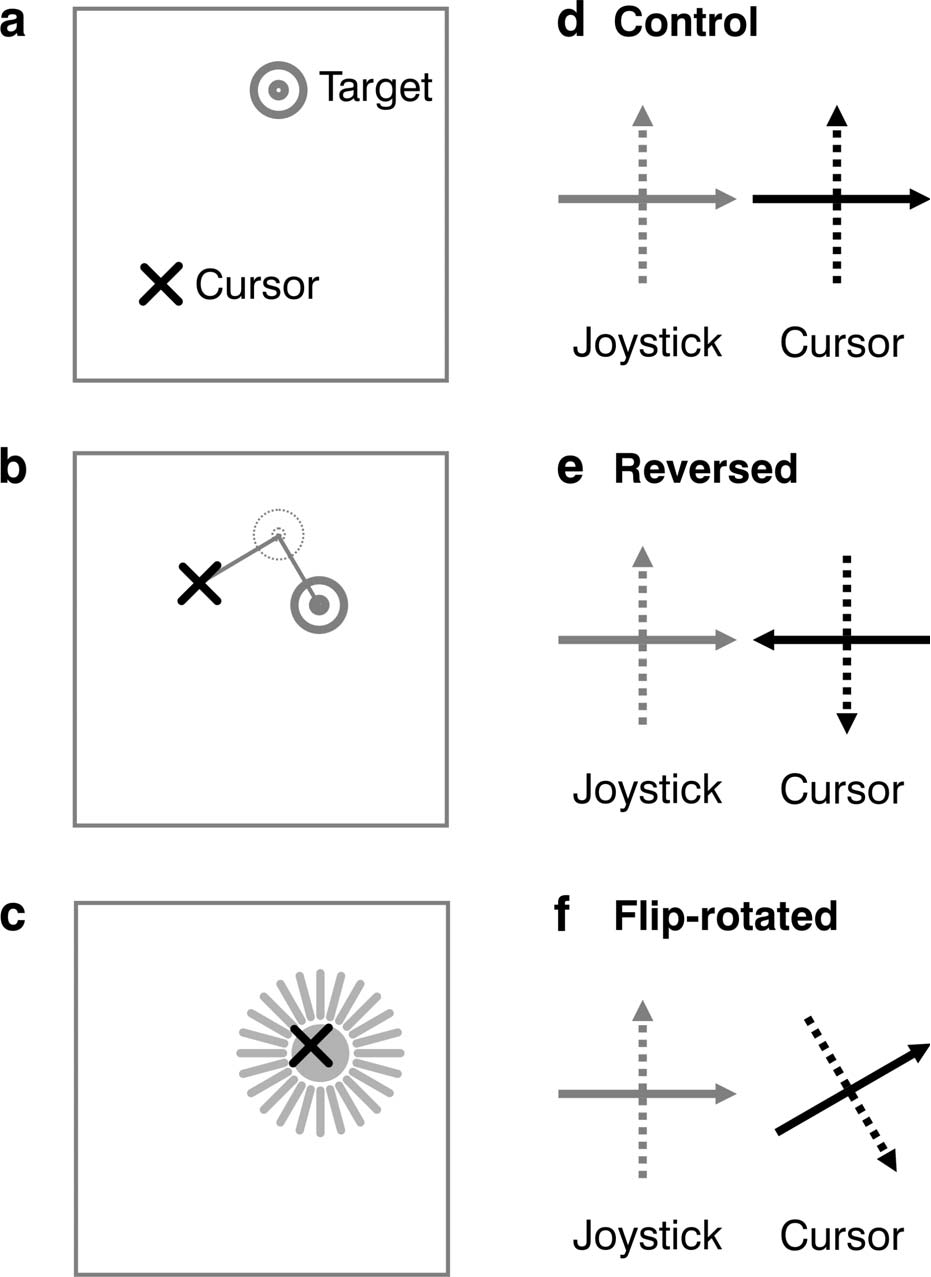
**Subjects bent a joystick to move a cursor to a target**. (**a**) In each trial, the target appeared at a random location. (**b**) The subject then moved the cursor, though often they didn't manage to move it exactly along a straight line to the target. During the cursor motion, the target jumped, at an unpredictable time, at right angles to the line from cursor to target. Jump size and direction were random. (**c**) If subjects got to the target and stayed there for 100 ms then they were rewarded with a flash and beep. (**d**) In control trials, pushing the joystick forward moved the cursor up, and pushing right moved it right (**e**) In Experiment 1, pushing the joystick forward moved the cursor down, and pushing right moved it left, i.e. cursor motion was reversed in both dimensions, flipping the signs of all components of ∂***e***/∂***u***. (**f**) In Experiment 2, the relation between joystick and cursor was more complex: reflected vertically through the midline and rotated 30 degrees counterclockwise.

On the screen were the cursor and target -- the cursor was an X, sized 0.5 cm by 0.5 cm, and the target was two concentric circles, 1 and 0.3 cm across. Cursor location was related to joystick angle by a linear mapping, with no velocity dependence. Subjects were instructed to bring the cursor to the target. At the start of each trial the target appeared at a random location within an *initial target range*, 13 cm by 13 cm, centered within a larger *movement range*, 13.75 cm by 13.75 cm, which was the range of motion of the cursor (the initial target range was smaller so that the target could always jump away from its initial point of appearance in an unpredictable direction and still remain within the movement range) (Figure 1a). The target's new location was computed from its previous one, at the end of the preceding trial, by the formula ***T***_*new *_= ***T***_*prev *_± random(5.5, 13) where random(5.5, 13) was a random vector, its two components chosen independently at the start of each trial from uniform distributions between 5.5 and 13; this random vector was added to ***T***_*prev *_on 50% of trials, and subtracted on the rest. Addition or subtraction was chosen randomly, except that if addition would have placed the target outside the initial target range then subtraction was used instead, and vice versa. In this way we kept the initial target inside its range but with plenty of variability and distance between successive appearances.

During each trial the target jumped once. Jump time was determined randomly, though based on cursor motion to help ensure that it occurred during the arm movement: the target jumped when ||***e***||, the size of the error vector ***e ***from target to cursor, first fell below a threshold value of random(0.25, 0.75)||***e***_0_||, where ***e***_0 _was the initial error vector when the target appeared at the start of the trial, and random(0.25, 0.75) was a number chosen at the start of each trial from a uniform distribution between 0.25 and 0.75. The *size *of the target jump was 60% as large as the error at jump time, i.e. 0.6||***e***||, except when a jump of that magnitude would have carried the target outside the movement range, in which case the *x-*and/or *y*-components of the jump were truncated to stay in range. The jump was orthogonal to the vector from target to cursor at the time of the jump (Figure 1b), except, again, when *x*- or *y*-components were truncated to stay in range. The *direction *of the jump, along this orthogonal line, was random: half the time in one direction, the rest in the other.

After the jump, subjects adjusted their motion to try to reach the target. If they managed to get the center of the cursor within 0.3 cm of the center of the target and hold it there for at least 100 ms within 2 s of the target's initial appearance then they were rewarded with a beep and a flash, i.e. the target changed momentarily from a pair of concentric blue circles to a filled-in red disk (Figure 1c). If they scored a beep, the next trial began immediately. If not, the next trial began 2 s after the initial appearance of the target. The initial cursor location, at the start of each new trial, was simply wherever the cursor happened to be at the end of the previous trial. Subjects saw the cursor and the target at all times throughout each trial, so they got plenty of feedback about their performance.

Subjects performed multiple blocks of 50 reaches. They had the option to rest as long as they liked between blocks. In control blocks, pushing the joystick forward moved the cursor up, and pushing right moved it rightward (Figure 1d). In test blocks, the relation between joystick and cursor was altered, in different ways in the two experiments described below, changing ∂***e***/∂***u***. On Day 1 subjects performed 20 blocks of 50 control reaches each, for 1000 reaches in all. On each of three or four subsequent days they did 20 blocks of reversed reaches, for a total of 3000 or 4000 reversed reaches. Finally, they did another 20 blocks of control trials. Through all these trials we sampled joystick position at 10-ms intervals.

### Experiment 1. Course adjustment with reversed sensitivity derivatives

**Figure 2:**
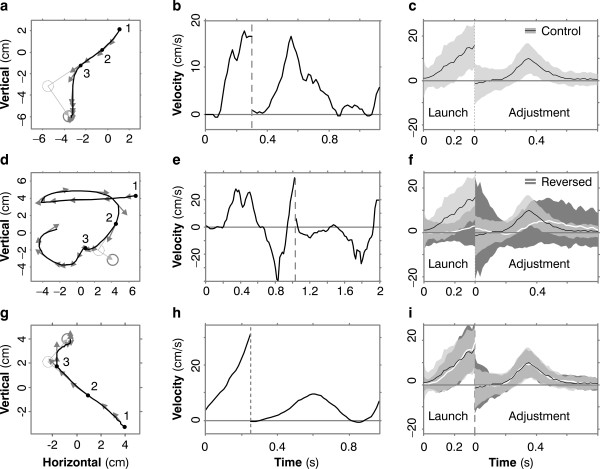
**Sample trajectories for one subject**. (**a**) A typical trajectory under control conditions (without reversed sensitivity derivatives). The cursor is at position 1 when the target appears (dotted gray circle). The cursor moves (black line) towards the target. Gray arrows are cursor-velocity vectors. When the cursor is at 2 the target jumps to its new location (solid gray circle). Near 3 the cursor adjusts course appropriately. (The markers 1, 2 and 3 have these same meanings in panels **d **and **g **also). (**b**) The same pattern is seen when we plot components of cursor velocity. Before the target jump (vertical dashed line), we plot cursor velocity in the direction from initial cursor location to initial target location; appropriately, this velocity is positive. After the jump, we plot cursor velocity in the direction of the jump, again appropriately positive. (**c**) The same pattern is seen in averaged velocity traces. Here we see cursor velocity -- mean (black line) and standard deviation (light gray band) over 200 randomly chosen control trials. Velocity is mainly positive, as it should be, during both launch and adjustment. (**d**) In early reversed trials, launch and course adjustments go the wrong way. (**e**) These errors are revealed also in velocity traces. (**f**) The white line and dark gray band are the mean and SD of cursor velocity over the first 200 reversed trials. As in Panel c, the black line and light gray band are the control data, and the light gray is transparent so that the dark gray data show through, for comparison. In the reversed trials, launch and adjustment are impaired, i.e. they are not consistently positive, and those portions that are positive occur later than in the control trials. (**g, h**) In *late *reversed trials, after the subject has learned, launch and adjustment are both correct. (**i**) Velocities averaged over 200 randomly chosen late reversed trials resemble controls.

**Figure 3:**
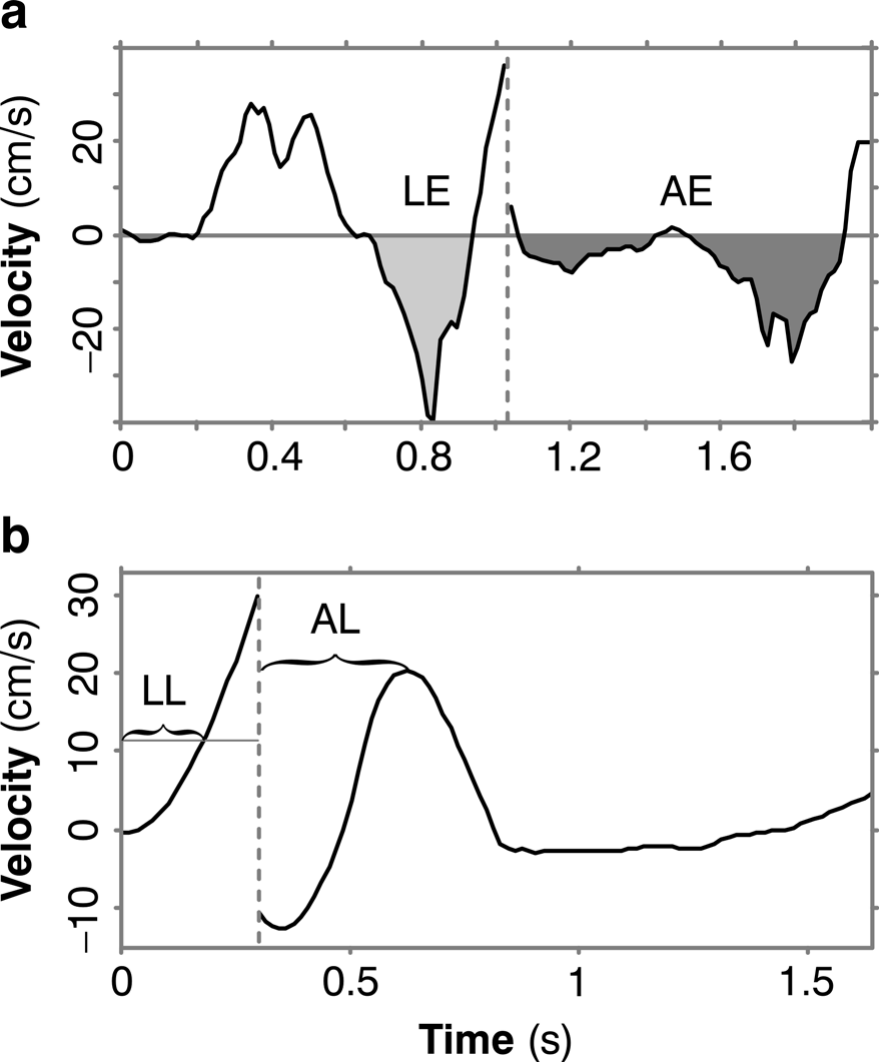
**Performance measures**. (**a**) Launch error (LE) is the area (colored light gray) above the negative parts of the cursor-velocity curve before the target jump. Adjustment error (AE) is the area (dark gray) above the negative parts of the curve after the jump. (**b**) Launch latency (LL) is the time from target appearance till cursor velocity exceeds the mean plus 3 times the standard deviations of initial velocity. Adjustment latency (AL) is the time from the jump till peak velocity.

**Figure 4:**
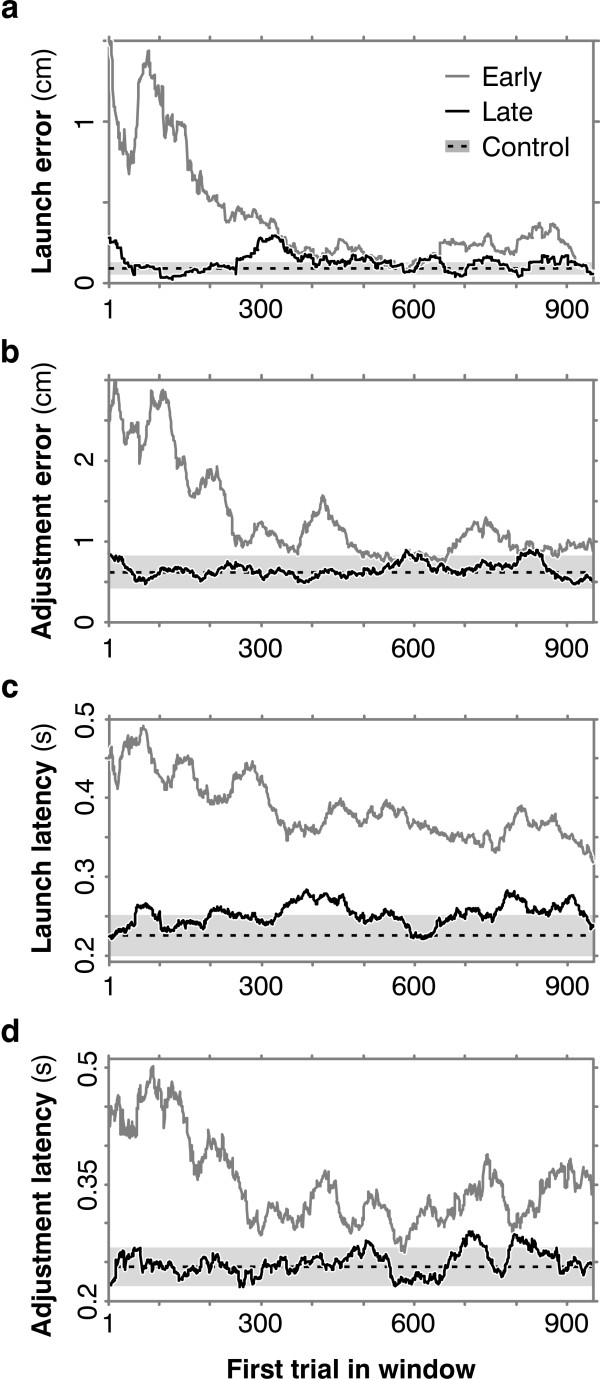
**Moving-window averages**. (**a**) To study changes through time we plot averages over a moving window of 50 trials, e.g. in this panel, the height of the curve above the 300 tick mark is the average launch error for trials 300-349; the rightmost point in the curve is the average for trials 951-1000. The plot shows that as the subject learns, launch error falls to control levels. The gray curve shows launch errors improving over this subject's first 1000 reversed trials. Black shows the final 1000 reversed trials. The light gray band is the mean ± 1 SD of the averaged launch error across all 950 windows in the initial control session. (**b**) Adjustment error also falls to control levels, as do (**c**) launch latency and (**d**) adjustment latency.

**Figure 5:**
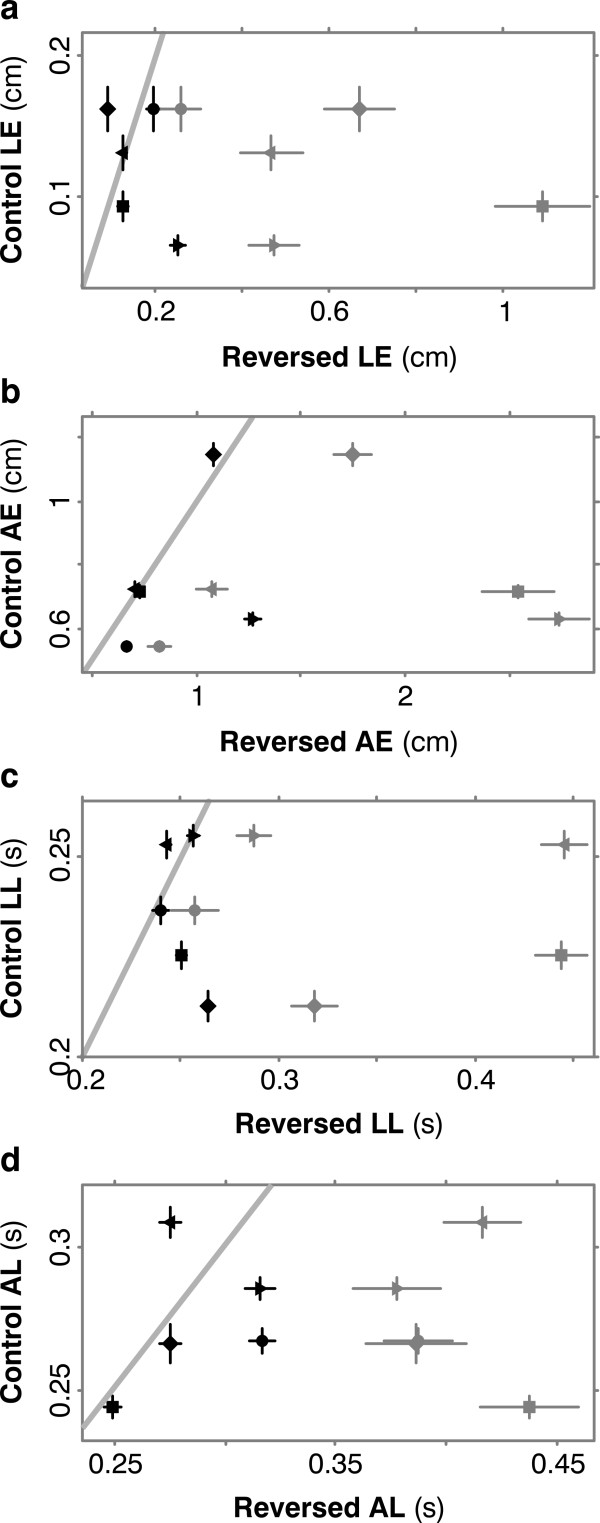
**All 5 subjects learned**. (**a**) On the ordinate are means and standard errors of launch error (LE) for the 1000 initial control trials. On the abscissa are the same measures for first 200 reversed trials (5 gray symbols for the 5 subjects) and for the last 1000 reversed trials (black symbols). Across all subjects, launch error improved significantly by t-test (p = 0.025) and in late reversed trials was not significantly different from control (p = 0.35). (**b**) Adjustment (AE) error also improved (p = 0.025) to control levels (p = 0.47). (**c, d**) Similarly for launch latency (LL) (p = 0.035 and 0.26) and adjustment latency (AL) (p = 0.004 and 0.43).

In test blocks, both dimensions of cursor motion were reversed from control, flipping the signs of all components of ∂***e***/∂***u ***(Figure 1e). Five subjects took part -- one female, four males, all healthy, aged 21-48. Three of them knew the experiment involved a reversed relation between joystick and cursor. One of these three had experience with joystick experiments, and one with joystick computer games. All our single-person data plots (Figure 2, 3, and 4) are of subjects who were unfamiliar both with joysticks and with the idea of motor adaptation to reversals, but the key findings were the same for all subjects, as shown in Figure 5.

### Experiment 2. Reversal and rotation

**Figure 6:**
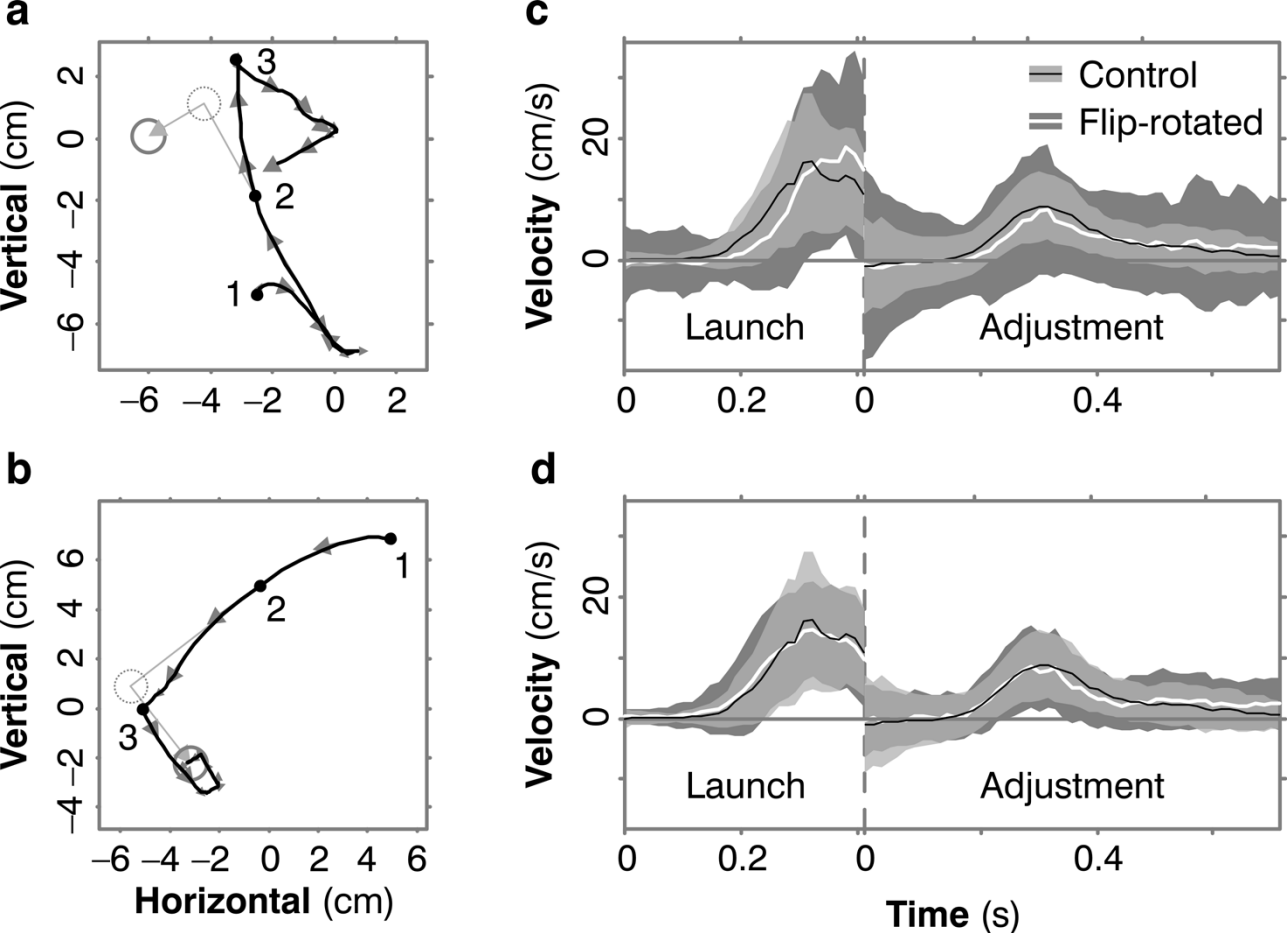
**Trajectories for one subject in Experiment 2**. Symbols are as in Figure 2. (**a**) In early flip-rotated trials, launch and adjustment go the wrong way. (**b**) In late flip-rotated trials, launch and adjustment are correct. (**c**) Averaged velocities in early flip-rotated trials are less consistently positive than in controls. (**d**) In late flip-rotated trials, they resemble controls.

**Figure 7:**
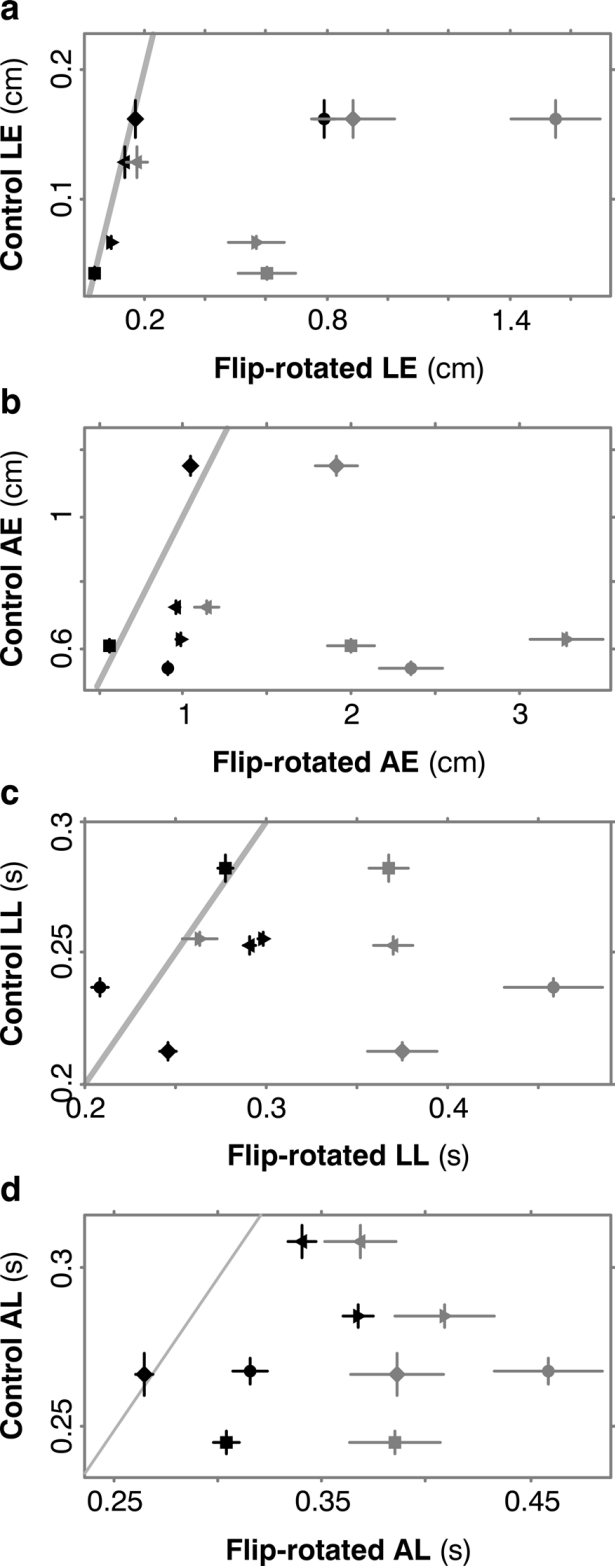
**Results for all 5 subjects in Experiment 2**. (**a**) Launch error improved by t-test (p = 0.008) to levels not significantly different from control (p = 0.339). (**b**) Adjustment error improved (p = 0.012) to control levels (p = 0.195). (**c**) Launch latency improved (p = 0.004) to control levels (p = 0.431). (**d**) Adjustment latency improved (p = 0.01), not quite to control levels (p = 0.036) but to within 80 ms of control.

Here the relation between joystick and cursor was more complex: reflected vertically through the midline and rotated 30 degrees counterclockwise (Figure 1f). Five subjects took part -- one female, four males, all healthy, aged 21-48. None of them knew the joystick-cursor relation beforehand. All found it bewildering, and none was able to state it afterwards based on their experience. Four of the subjects were veterans of Experiment 1, and therefore had more joystick experience in this second part, but that fact is irrelevant here because our hypothesis and analysis involved no comparisons of the two experiments. The single-person data plot (Figure 6) is of the new subject, without joystick experience, but the key results were the same for all, as shown in Figure 7.

## Results

### Control trials

In control trials, subjects' course adjustments usually go the right way. Figure 2a shows a typical movement: the cursor moves off in the direction of the initial target; after the target jumps, the cursor adjusts in the direction of the jump. The same pattern is seen in a plot of cursor velocity for the same movement, Figure 2b. In this figure and all other velocity-versus-time plots in the paper, we show different components of the cursor-velocity vector before and after the jump (marked by the vertical dashed gray line): before the jump we plot the component of cursor velocity in the direction from initial cursor location to initial target location; after the jump we plot the component in the direction of the jump. Graphed this way, positive velocity in the first stage of the plot indicates that the cursor has launched appropriately, in the direction of the target, and positive velocity in the second stage means the cursor has adjusted appropriately to the jump. In Figure 2b, both components are mainly positive.

### Experiment 1: Course adjustments with reversed sensitivity derivatives

In *early *reversed trials, both launch and course adjustment go the wrong way, as shown in Figure 2d. The errors are revealed also in the velocity trace for the movement, Figure 2e: before the target jump, cursor velocity is not consistently positive, i.e. not in the direction of the target; after the jump, cursor velocity is mostly negative, i.e. opposite the jump. These plots also show that, even in early reversed trials, subjects don't move relentlessly in the wrong direction, but rather their trajectories are confused, with a tendency to go the wrong way and then try to correct. But after the subject has performed 3000 trials under reversed conditions, launch and adjustment are both appropriate, as shown in Figure 2g and 2h. This is the key result of our study: movement traces like these show that the subject, after training, could make online course adjustments with no wrong-way response, as predicted by the theory of implicit supervision.

To show that this behavior was consistent, we averaged cursor velocities over many trials. Figure 2f and 2i show mean velocity and its standard deviation for one subject, with control data superimposed on the data for trials with reversed sensitivity derivatives. In control traces (Figure 2c), cursor velocity is appropriately positive in both the launch and adjustment stages of the movement. In early reversed trials (Figure 2f), velocities are not consistently positive during launch or adjustment. In *late *reversed trials (Figure 2i), velocities are again appropriate, and resemble controls as regards direction, size, timing, and variance. This same result was seen in all five subjects.

To chart these improvements through time, we plotted four performance measures. We quantified wrong-way responses by integrating negative velocity, as shown in Figure 3a. We defined the *launch error *(LE), for any one trial, to be the area above the negative parts of the cursor-velocity curve before the target jump (light gray region), i.e. launch error is the distance traveled away from the target over this period. *Adjustment error *(AE) is the area above the negative parts of the velocity curve *after *the jump (dark gray region), i.e. it is the distance traveled opposite the jump. To quantify timing, we defined *launch latency *(LL) to be the interval from the target's initial appearance until positive cursor velocity (the component in the direction from initial cursor position to initial target) exceeded a threshold value *v_th_*, where *v_th _*was equal to the mean plus three times the standard deviation of the subject's cursor velocity at target appearance, averaged across all control trials for that subject. *Adjustment latency *(AL) is the time it takes from the target jump until cursor velocity (the component in the jump direction) reaches its first maximum at least 150 ms after the jump (Figure 3b). So adjustment latency indicates how quickly a given subject on a given trial produced an appropriate course adjustment. It overestimates reaction time, because (for robustness) it is based on the peak, rather than the onset, of the right-way velocity. But it provides a consistent measure of the time frame of our subjects' course adjustments.

Figure 4 shows moving-window plots for all four measures -- launch error, adjustment error, launch latency, and adjustment latency -- for one subject. Each of the curves shows one performance measure, averaged over a 50-trial moving window, through one session of 1000 trials. In each panel, the jagged gray line shows the error or latency improving over the first 1000 reversed trials; the light gray band shows the mean and one standard deviation for the same error or latency over the 1000 initial control trials; and the jagged black line shows performance in the final 1000 reversed trials. In all four panels, performance over the last 1000 trials hovers near the control range. Again, the plots were very similar for all five subjects.

Figure 5 summarizes the results for these four measures for all subjects. Means and standard errors are plotted for the 1000 control trials on the ordinates and for reversed trials on the abscissas, with early and late trials in different colors: the five gray symbols -- one for each subject -- mark the means and standard errors over the first 200 reversed trials; black symbols show the data for the final 1000 reversed trials. Slanted gray lines indicate where values for control and reversed performance are equal. In all four plots, all five subjects improved: the black symbols lie to the left of the gray ones, and significantly at p < 0.05 by t-test and sign test (a non-parametric test for paired samples, [18]).

Further, in all four plots there was *no *significant difference, at the same p level, between control and late reversed trials, i.e. subjects returned to something like control performance. This finding is interesting but peripheral to our purposes, because the theory of implicit supervision doesn't imply anything about whether post-adaptation performance will be identical with controls. The point of Figure 5 is that all subjects improved, driving down their adjustment errors without appreciably slowing their responses.

Three subjects knew the experiment involved a reversed relation between joystick and cursor. The other two subjects never recognized the relation, i.e. they couldn't state it in words when questioned after the experiment was over. None of the five subjects felt, introspectively, that it helped to try to work out the relation of cursor to joystick, or to imagine the target in some reversed location on the computer screen, or to reverse their hand motion deliberately. What worked was simply to chase the target with the cursor, giving no thought to hand motion, improving gradually and automatically.

### Experiment 2: Reversal and rotation

In this harder task, the results were like those in Experiment 1 except that learning was slower. Plots of individual movements showed that in early flip-rotated trials, launch and adjustment were both inappropriate (Figure 6a), but in late trials both had adapted (Figure 6b). Averaged velocity traces showed that this pattern was consistent (Figure 6c, d). Across subjects, all four performance measures improved significantly with training, to near-control levels (Figure 7). None of the subjects, even after learning the task, was able to state in words the relation between joystick and cursor.

## Discussion

The theory of implicit supervision holds that the brain's estimates of sensitivity derivatives, ∂***e***/∂***u***, can be revised based on sensory feedback [4,5,13]. This theory explains how neural controllers can deal with sign changes in ∂***e***/∂***u***. For instance, humans and monkeys can learn to handle objects and navigate while wearing reversing prisms [3,15,16]. People can learn to mirror-draw, and dentists can drill teeth seen in a mirror. When antagonist muscles or nerves are transposed, animals can sometimes regain their coordination [6-11,19]. And facial-palsy patients treated by hypoglossal nerve transposition learn to control face and tongue independently [12,14,20]. The theory also explains why it is harder to adapt to reversals than to other changes: displacing, magnifying, and minifying goggles don't flip the signs of ∂***e***/∂***u***, so we can adapt to them without revising our estimates of the sensitivity derivatives; reversing prisms, on the other hand, do flip the signs, so we can't adapt without re-estimating ∂***e***/∂***u***.

Here we have confirmed another prediction of the theory: in arm-movement tasks with reversed and rotated sensitivity derivatives, our subjects learned to make appropriate course adjustments when the target jumped. After training, individual movements often showed no wrong-way response (naturally some movements did show mistakes, during launch or adjustment, but similar mistakes were seen also in control trials). Averaged velocity traces in the reversed task after training resembled control traces as regards direction, size, timing, and variance. For all subjects, wrong-way responses shrank (as quantified by adjustment errors), to near control levels. So the neural estimate of ∂***e***/∂***u ***that is used for course adjustments is clearly revisable.

How do our results fit with those of Gritsenko and Kalaska [17]? For our purpose -- testing implicit supervision -- what is important about that study is the discrepancy between launch and adjustment: some of the subjects who learned to launch toward the target still made course adjustments in the wrong direction. That finding raised a question for our theory: if the subjects improved their launches by re-estimating their sensitivity derivatives then why didn't the revised estimate correct their adjustments?

One possible explanation is that there are two (or more) separate estimates of ∂***e***/∂***u ***for different aspects of a task, e.g. for launch and adjustment. In this view, a subject might reverse their launch-related estimates of ∂***e***/∂***u ***but not their adjustment-related estimates, maybe because the latter change more slowly, or because the training included no practice adjusting to target jumps. (A different issue is whether launch and adjustment involve separate controllers, e.g. one using feedback and the other not. This is a separate question because even entirely disjoint launchers and adjusters, whether feedback-guided or not, could still be governed by a single estimate of ∂***e***/∂***u***. Our concern here is with ∂***e***/∂***u***, not with other possible contrasts between launching and adjusting.)

But a simpler explanation is that there is one all-purpose estimate of ∂***e***/∂***u***, and Gritsenko and Kalaska's subjects revised it over only part of its domain. The key point is that ∂***e***/∂***u ***is not a constant matrix but varies over a domain *D*. For instance we might have *D *= *X *× X* × *U*, where *X *is the state space of the plant (e.g. the space of all possible combinations of arm joint angles and velocities), X* is the space of target states, *U *is the space of motor commands, and × is the Cartesian product. When a target jumps during an arm movement, it suddenly transports the subject to a new region of *D*. (In Gritsenko and Kalaska's experiment, subjects may not have been transported very *far *through *D*, as the target jumped only 10°, measured from the starting point of the movement. But by the time they reacted, their angular errors would be larger than 10°. And even if the new region of *D *were close to the old, the appropriate motor command might be quite different there, as the subjects would need lateral acceleration in situations where the target had jumped.) Gritsenko and Kalaska's study was designed to train people with no jumps and then test their generalization to jumping targets, so their subjects had little experience with the post-jump regions of *D*, and therefore, we suggest, didn't completely revise their estimates of ∂***e***/∂***u ***there; some learning may have generalized from nearby regions, but not enough to abolish their inappropriate, unreversed responses. Our study was designed to give subjects plenty of experience with jumps during their training, and so they learned ∂***e***/∂***u ***over the relevant parts of *D*.

This idea doesn't imply that there are "boundaries" within *D*, or that different regions of it are linked with different learning mechanisms or controllers. The point is simply that a learner trained in one domain usually does poorly in others, e.g. a neural network trained to approximate the function *x*^2 ^over the domain [0, 0.1] does poorly when tested over a different region, say [0.1, 0.2]. And the failure is worse, the more the target function differs between the two regions. Similarly, implicit supervision trained exclusively on one subset of *D *-- the subset inhabited by reaches to fixed targets -- yields poor estimates of ∂***e***/∂***u ***elsewhere.

The four types of learning curves in Figure 4 -- launch error, adjustment error, launch latency, and adjustment latency -- decline with roughly similar time courses. Unfortunately their shapes offer no clues as to how many estimates of ∂***e***/∂***u ***are being adapted. The similarity between the four curves need not imply a single estimate of ∂***e***/∂***u ***underlying them all; it is also compatible with multiple estimates of ∂***e***/∂***u ***if those estimates learn in similar ways. And conversely, even markedly dissimilar curves would be compatible with a single estimate of ∂***e***/∂***u ***because the four curves reflect different aspects of the task, occurring in different regions of the domain *D*. They are expected to differ, even if they all depend on the same estimate of ∂***e***/∂***u***. In simulations, the correlations and other similarities between these curves vary enormously depending on assumptions about learning algorithms, neural coding, and noise throughout the control system, i.e. both single and multiple estimates are compatible with a wide variety of curves.

In both our experiments, subjects' responses were often delayed, e.g. in Figure 5 and 7, LL and AL were always greater in early reversed trails than in control trials, and often stayed greater for thousands of trials, though eventually they improved to roughly control values. Evidently subjects slowed some aspects of their movements in unfamiliar conditions, maybe to permit more voluntary control.

Voluntary reversals have been studied by Day and Lyon [21]. Their subjects reached straight ahead for a target which jumped right or left in mid-reach. The subjects were told to react to the jump by moving in the opposite direction, but even after several hundred trials, their first reaction was still in the jump direction, followed by a reversed response. What does this mean for implicit supervision? There are many possibilities, e.g. 1) Day and Lyon's results may have nothing to do with changes in ∂***e***/∂***u***. Their study involved no sensory reversal, so there was no change in the relation between any sensory error signal ***e ***and motor commands; rather there was a verbal instruction to reverse. Subjects may simply have tried to aim for an imaginary target opposite the real one, in keeping with their instructions. 2) Subjects may have created a new, mental error signal ***e***' equal to -1 times the visual error ***e***, and then learned ∂***e***'/∂***u***. They may have had two separate representations of ∂***e***'/∂***u ***for early and late responses to jumps. Or their early and late responses may have been guided by ∂***e***/∂***u ***and ∂***e***'/∂***u ***respectively. 3) Subjects may have had one representation of ∂***e***'/∂***u ***for reflexive control generally and another for higher-level control, i.e. separate representations for different levels of control rather than for different stages of a movement.

There may be hints of multilevel control in our results as well, e.g. in Figure 1d an early reversed trial, the subject launches in an inappropriate direction but then later, something makes them reverse course with a tight U-turn (though the new direction is also inappropriate). If there is a high-level controller that steps in here, it may have a separate estimate of ∂***e***/∂***u***, better than the reflexive controller's, but this scheme would be inefficient: learning ∂***e***/∂***u ***is computationally costly, so there are good reasons to do it just once. Another possibility is that the high-level controller has no good estimate of ∂***e***/∂***u***, but adopts some simple, exploratory strategy, e.g. it thinks "my estimate of ∂***e***/∂***u ***is clearly inaccurate, and my most recent action was counterproductive, so I'll try undoing it or doing something else different". Or maybe high-level controllers can rapidly estimate the current value of ∂***e***/∂***u***, i.e. they don't learn the *function *∂***e***/∂***u ***but just estimate its *value at the current spot *in its domain *D*, which is easier. This approach would bring advantages if used to supplement (not replace) learning the function ∂***e***/∂***u ***-- see Fortney and Tweed [22].

Where in the brain might ∂***e***/∂***u ***be represented? One possibility is the cerebellum, which is involved in sensorimotor learning and internal models [23]. These models are neural circuits that mimic aspects of the system to be controlled, such as the mechanical properties of an eyeball or limb, and especially the relation between neural commands and motor performance. In particular, so-called forward models mimic the response of the controlled system to neural commands [23]. Therefore an estimate of ∂***e***/∂***u ***is a kind of forward model, representing the relation between performance error ***e ***and command ***u***.

## Conclusions

We have shown that people can learn to reverse their online course adjustments, implying that these adjustments are based on revisable estimates of sensitivity derivatives, as predicted by the theory of implicit supervision. And we have argued that the available data are consistent with the simplest version of the theory, that a single, contextual estimate of ∂***e***/∂***u ***guides motor learning for all stages of a task, including launch and adjustment.

## Competing interests

The authors declare that they have no competing interests.

## Authors' contributions

MA helped design the study, conducted the experiments, and contributed to data analysis, text, and figures. DT helped design the study and contributed to data analysis, text, and figures. Both authors read and approved the final manuscript.
